# Evaluation of Phytochemistry, Toxicity and Radical-Scavenging Capacity of Two *Erigeron* Species (*E. annuus* (L.) Pers. and *E. strigosus* Muhl. ex Willd.) Extracts

**DOI:** 10.3390/plants15182851

**Published:** 2026-09-18

**Authors:** Asta Judžentienė, Jurga Būdienė

**Affiliations:** 1 Center for Physical Sciences and Technology, Department of Organic Chemistry, Sauletekio Avenue 3, LT-10257 Vilnius, Lithuania; jurga.budiene@ftmc.lt; 2 Institute of Biosciences, Life Sciences Center, Vilnius University, Saulėtekio Avenue 7, LT-10257 Vilnius, Lithuania

**Keywords:** *Erigeron annuus* (L.) Pers., *Erigeron strigosus* Muhl. ex Willd., extracts, polyphenols, HPLC/DAD/TOF, toxicity, *Artemia* sp., ABTS and DPPH radical-scavenging potential

## Abstract

*Erigeron* sp. has been employed for medicinal purposes in the ethnopharmacology of numerous countries worldwide, as well as in modern-day herbal applications. Their healing capacity is determined by their bioactive compound content, with polyphenols playing a predominant role. Phytochemistry of two alien species in Lithuania, *E. annuus* (L.) Pers. and *E. strigosus* Muhl. ex Willd. was investigated. The phytochemicals in the extracts of flowers, leaves and roots were identified by the HPLC/DAD/TOF technique. Some acids, such as fumaric, succinic, quinic, malic, protocatechuic, *p*-coumaric, gelseminic and (iso-, neo-) chlorogenic, were found in the extracts of both investigated *Erigeron* species. Additionally, flavonoids and other constituents were tentatively identified in the plant extracts. Umbelliferone, loliolide, kaempferol/luteolin, erigeside I, baicalin, quercetin and its glucoside, apigenin and its glucuronide, apigenin-7-*O*-glucuronide-6′-ethyl ester, astragalin/quercitrin, scutellarin, caffeoylshikimic acid glucoside, sesquiterpene hydrocarbon(s) and rutin were found to be common compounds in all extracts. A toxicity test using brine shrimp *Artemia* sp. larvae revealed that the activity order between both fleabane organs was as follows: leaves > flowers > roots. *E. strigosus* extracts exhibited slightly stronger toxic effects than *E. annuus*. Total phenolic content (TPC) varied from 11.88 ± 1.27 to 304.94 ± 3.81 (mg/L, expressed in gallic acid equivalent (GAE)) in aqueous *E. annuus* root and methanolic *E. strigosus* leaf extracts, respectively. TPC and antioxidant ability differed significantly between the plant organs of both fleabanes. ABTS^●+^ and DPPH^●^ scavenging capacity was detected for aqueous extracts of both *Erigeron* species, ranging from 0.16 ± 0.02 (ABTS^●+^) to 17.55 ± 0.07 (DPPH^●^) (mmol/L, TROLOX equivalent) for *E. strigosus* roots and *E. annuus* leaves, respectively. A strong positive correlation between TPC and ABTS^●+^ scavenging capacity was determined (*p* = 0.01996 and *p* = 0.01171 for *E. annus* and *E. strigosus* extracts, respectively), while the correlation between TPC and DPPH^●^ assay values was also positive, but did not reach statistical significance (*p* > 0.05). For the first time, the data concerning the phytochemistry, toxicity and radical-scavenging capacity of *E. strigosus* have been documented. The present study makes a substantial contribution to studies already existing on bioactive phytochemicals of *E. annuus*, with a particular focus on *E. strigosus* plants.

## 1. Introduction

Two species of fleabane were investigated in the research, namely *E. annuus* (L.) Pers. (Desf.) and *E. strigosus* Muhl. ex Willd. (Bigelow), which are attributed to the genus *Erigeron* (f. *Asteraceae*) along with more than 450 others [1,2].

**Figure 1:**
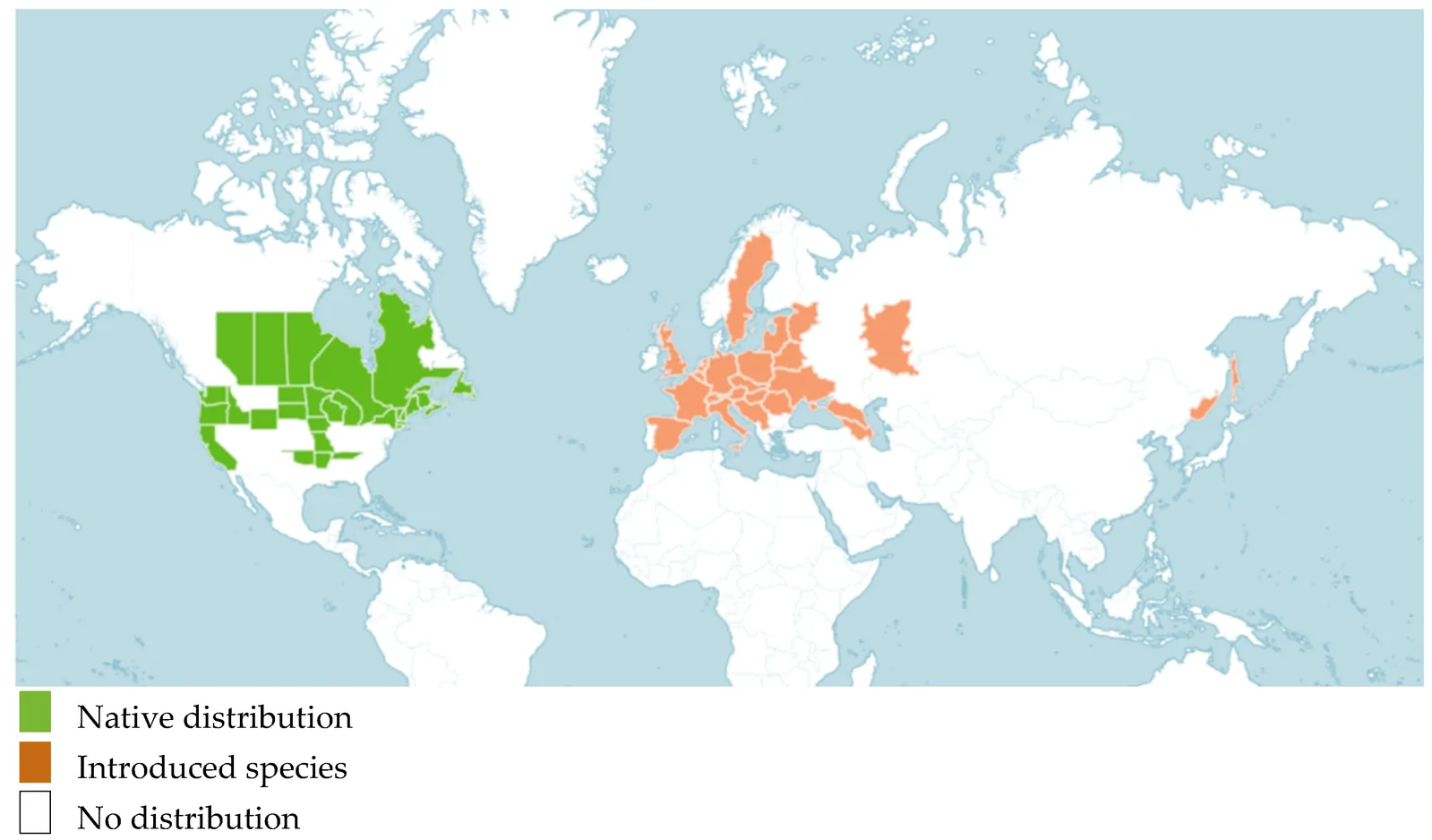
An illustration of the worldwide distribution of *Erigeron annuus* (L.) Pers. plants. The image was created by applying data from Plants of the World Online (POWO) [2] and the Canva design platform (https://www.canva.com).

*E. annuus* (L.) Pers., otherwise known as daisy fleabane and annual fleabane, is categorized as an annual herb, although it may sporadically grow as a biennial, with growing habitats primarily in the temperate biome; it typically produces flowers with white petals and yellow centres [2]. This species is native to North America, spanning from Eastern Canada to the Central and Eastern USA [2,3]. It is considered to be a medicinal and aromatic plant in indigenous habitats [4]. Annual fleabane exhibits numerous biological features that enable its capacity for widespread growth, inhabiting a broad spectrum of environmental conditions. According to data from the Global Biodiversity Information Facility (GBIF [5]), *E. annuus* (L.) Pers. has been introduced into 43 countries or islands, and the most prevalent distribution has been recorded in Switzerland and France, with 153,280 and 62,900 occurrences, respectively [3]. A limited number of growing habitats are indicated in Costa Rica, Asian countries (Kazakhstan, Japan, China, Nepal, Korea, India, Vietnam, etc.), and some islands, such as Newfoundland, Ireland, Corse, Sicilia, Réunion, Kuril, etc. [2,3]. Up to 6 July 2022, 325 occurrences of annual fleabane were identified in Lithuania [3], where it is considered an invasive species [6,7]. However, the annual fleabane poses a considerable threat to the native plants of numerous countries as well as to agricultural activities. A global occurrence of *E. annuus* (L.) Pers. (Desf.) plants is shown in Figure 1.

**Figure 2:**
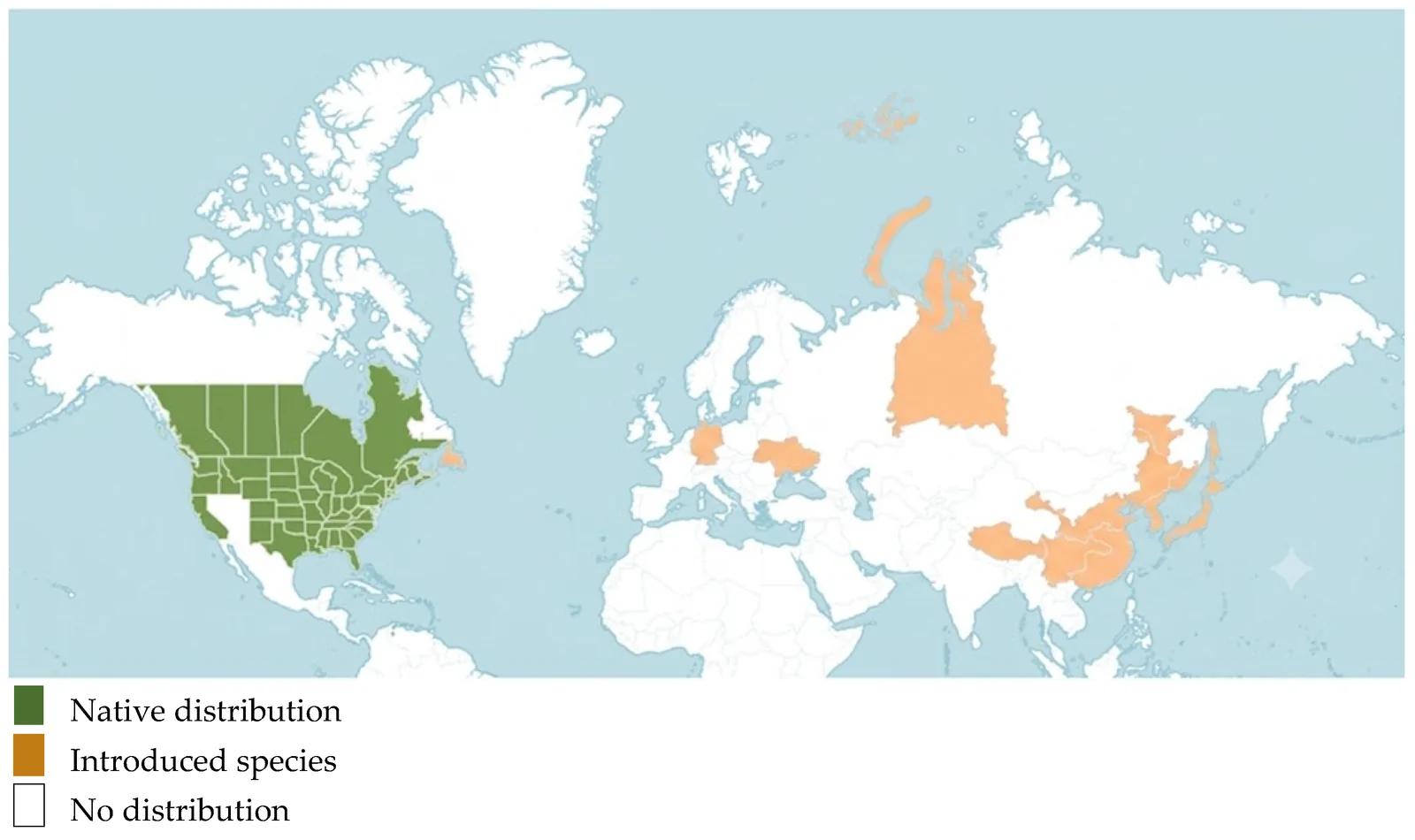
A visual indication of *Erigeron strigosus* Muhl. ex Willd. global occurrence. The picture was created using the POWO (Plants of the World Online) database [2] and the Gemini software (Gemini Web Application) (https://gemini.google.com/app (accessed on 4 June 2026)).

*E. strigosus* Muhl. ex Willd. is known by common names, like common eastern fleabane, rough fleabane, prairie fleabane, or vergerette rude; and at least 33 homotypic synonyms of this species have been reported in the international networks [2,3]. As the rough fleabane (*E. strigosus*) is morphologically very similar to the annual fleabane (*E. annuus*), it has had synonymic names such as *Erigeron annuus* subsp. *strigosus* (Muhl. ex Willd.) Wagenitz; *Erigeron annuus* var. *ramosus* (Walter) Hyl. or *Erigeron ramosus* var. *discoideus* Britton, Sterns & Poggenb [2,3]. *E. strigosus* can be distinguished from *E. annuus* by the fact that all of *E. strigosus’* parts are covered in close-lying hairs that are antrorse strigose, and its achenes are smaller, with shorter ligulate flowers. Rough fleabane is a plant that is characterized by its basal leaves; white, bluish or pinkish flowerets; an erect or ascending stem that is tall (30–80 cm) and covered in many fine white hairs; and the fibrous root system. *E. strigosus* is a shrub that can be found in different growth forms: as an annual, biennial or short-lived perennial, and has the ability to produce a multitude of flower heads [8]. Flora of North America (FNA) recognizes four distinct varieties of *E. strigosus*: *septentrionalis*, *strigosus*, *dolomiticola* and *calcicole* [8]. Rough fleabane, being native to Canada and the USA, grows in temperate regions [9]. Nonetheless, the species is regarded as an alien plant in numerous regions across Asia, including West Siberia, Tibet, China, Korea, Japan and Sakhalin, as well as in various parts of Europe [2]. GBIF recorded *E. strigosus* as an introduced species in 14 countries or islands [3]. The largest number of occurrences (1328) of *E. strigosus* were documented in Romania, whereas the species was only registered in five growing locations in Lithuania from 2013 to 2024 [3]. *E. strigosus* Muhl. ex Willd. has been recorded as an alien weed in Lithuania [7,10]. The worldwide spread of *E. strigosus* Muhl. ex Willd. plants is demonstrated in Figure 2.

Historically, indigenous North Americans have used rough fleabane for various medicinal purposes, primarily as an anti-inflammatory and digestive remedy [11], and preparations of annual fleabane have been used against tuberculosis, diarrhea, dysentery, to treat coughs and respiratory illnesses, cardiovascular problems, reduce or stop bleeding, etc. [12]. *E. annuus* has a long history in traditional Chinese medicine, where it is widely used in diverse formulations for the treatment of various ailments, including malaria, enteritis, indigestion, hepatitis, diabetes and obesity [12]. A literature review of *E. annuus* (L.) phytochemistry [12,13,14,15,16,17,18,19,20,21,22,23,24,25,26,27,28,29,30,31,32,33,34,35,36,37,38,39,40] revealed that various extracts of the plant contain a huge variability of pharmacological important compounds, such as terpenoids [12,13,14,15,16,17,18], phenolic and organic acids [12,13,18,19,20,21,22,23,24,25,26,27,28,29], flavonoids [12,18,19,22,23,25,26,27,29,30,31,32,33,34], lactones [12,15,16], coumarins [12,18], sterols [12,17,18,35], polysaccharides [26,36,37], amino acids [38], etc.

Numerous modern scientific studies have revealed evidence of the many ethnopharmacological applications, indicating that extracts or active compounds derived from *E. annuus* possess antioxidant properties [12,19,22,24,25,26,34,41,42], anti-inflammatory effects (through NF-κB inactivation [43,44] and heme oxygenase-1 induction [41,45]) [12,41,43,44,45]; this includes protein glycation inhibitory (against cataractogenesis, aldose reductase and formation of advanced glycation products) [20,21,24,30], neuroprotective [12,19], anti-obesity [12,27,42], antidiabetic (alleviation of insulin resistance) [28], anti-atherosclerotic [12,35], antiproliferative and anti-fungal [12,13,16], cytoprotective [31,41], anti-tumour (to human cells of leukemia (HL-60), hepatoma (SMMC-7721) and embryo liver (L-02)) [39], and antiviral (against the respiratory syncytial virus) [32] activity. However, germination inhibitory effects and allelopathic properties (on major crop species) were revealed for *E. annuus* [23,46].

To the best of our knowledge, data in the literature related to the phytochemistry and bioactivities of *E. strigosus* are very limited [47,48,49,50,51,52]. Crude organic extracts of rough fleabane showed remarkable antimicrobial activity against *Mycobacterium tuberculosis* H_37_Rv and *Mycobacterium avium* (using the BACTEC 460 radiorespirometric assay) [48]. Among the ethnomedicinal properties of wild plants from Pakistan (South Punjab), *E. strigosus* preparations (juice, decoctions, powder and tea) were documented as a remedy against fever, liver disorders, skin issues and as a blood purifier [49]. Due to anti-inflammatory and analgesic properties, Aboriginal people of the Canadian boreal forest have used rough fleabane to treat chronic pain syndromes of headache and migraine [50]. No sensitization was revealed for *E. strigosus* using patch tests in order to detect contact allergy and/or dermatitis [51]. Ethanolic extracts of rough fleabane (including leaves, flowers, stems and fruits) collected in the USA did not show any antimicrobial effects against *C. albicans, E. coli*, *S. aureus* and *P. aeruginosa* [52].

Both species, *E. annuus* (L.) Pers. and *E. strigosus* Muhl. ex Willd. are considered alien plants in Lithuania. *E. annuus* is also an invasive plant. Due to its aggressiveness, the alien species is able to oust other plants from their native growing localities, thereby reducing biodiversity and altering the entire ecosystem. Knowledge of the phytochemistry of non-indigenous flora is important for two primary reasons: firstly, it helps us to control their aggressive propagation, and secondly, it enables the development of bioherbicides based on the phytotoxic compounds present in them.

The objective of the study was to facilitate a comparison of two alien *Erigeron* species with the following specific aims: (i) to evaluate the phytochemical composition of different plant organs (flowers, leaves and roots, and during the full flowering stage) of *E. annuus* (L.) Pers. (annual fleabane) and *E. strigosus* Muhl. ex Willd. (rough fleabane and herbal material collected over two years from the same growing habitat, limited in area) extracts; (ii) to reveal their preliminary toxicity by in vivo tests, using larvae of *Artemia salina* brine shrimps; (iii) to determine total phenolic content (TPC) in the extracts; and iv) to determine their antioxidant potential employing the spectrophotometric radical (ABTS^●+^ and DPPH^●^) scavenging assay.

## 2. Results

### 2.1. Chemical Composition of Erigeron annuus (L.) Pers. (Annual Fleabane) Extracts

At least 36 compounds (among them 13 phenolic acids) were identified tentatively by DAD and TOF (Table 1) in aqueous and methanolic (MeOH:H_2_O, 1:1, *v*/*v*) extracts from various organs (inflorescences, leaves and roots) of *E. annuus* plants.

Eleven constituents, such as (neo)chlorogenic and isochlorogenic (A and B) acids, umbelliferone, matricaria lactone, erigeroflavanone, baicalin, apigenin-7-*O*-glucuronide, scutellarin and quercetin 3′-*O*-glucoside, were detected by both (positive and negative) ionizations. The rest of the compounds provided *m*/*z* ions only in a positive or negative ionization mode.

### 2.2. Chemical Composition of Erigeron strigosus Muhl. ex Willd. (Rough Fleabane) Extracts

In total, at least 45 compounds (among them 18 acids) were detected in *E. strigosus* inflorescence, leaf and root extracts (both aqueous and methanolic (MeOH:H2O, 1:1, *v*/*v*)) by DAD and TOF (in positive or negative ionization mode) (Table 2).

*p*-Coumaric; linolenic; 9,12,13-trixydroxy-10*E*-octadecenoic; chlorogenic, neo- and isochlorogenic (A and B) acids; lachnophyllum ester; 5,7-dihydroxychromone; loliolide; baicalein; apigenin; (epi)catechin; quercetin; quercetin 3′,4′,7-trimethyl ether; erigeside I; baicalin; apigenin-7-*O*-(methyl)glucuronide; quercetin 3′-*O*-glucoside and (-)-syringaresinol-4-*O*-*β*-D-glucopyranoside were detected under both negative and positive ionization conditions. The remaining constituents provided *m*/*z* ions only in one (positive or negative) ionization mode.

### 2.3. Toxic Activity of Erigeron annuus (L.) Pers. and Erigeron strigosus Muhl. ex Willd. Aqueous Extracts

A toxicity test, engaging brine shrimp *Artemia salina* (L.) (Anostraca: Artemiidae) larvae and aqueous extracts of *E. annuus* and *E. strigosus,* revealed that the activity order between fleabane organs is as follows: leaves > flowers > roots (Table 3). Leaf extracts of both *Erigeron* species exhibited similar toxicity. Rough fleabane flower and root extracts were slightly more toxic than annual fleabane corresponding extracts.

### 2.4. Total Phenolic Content (TPC) of Erigeron annuus (L.) Pers. and Erigeron strigosus Muhl. ex Willd. Extracts

**Figure 3:**
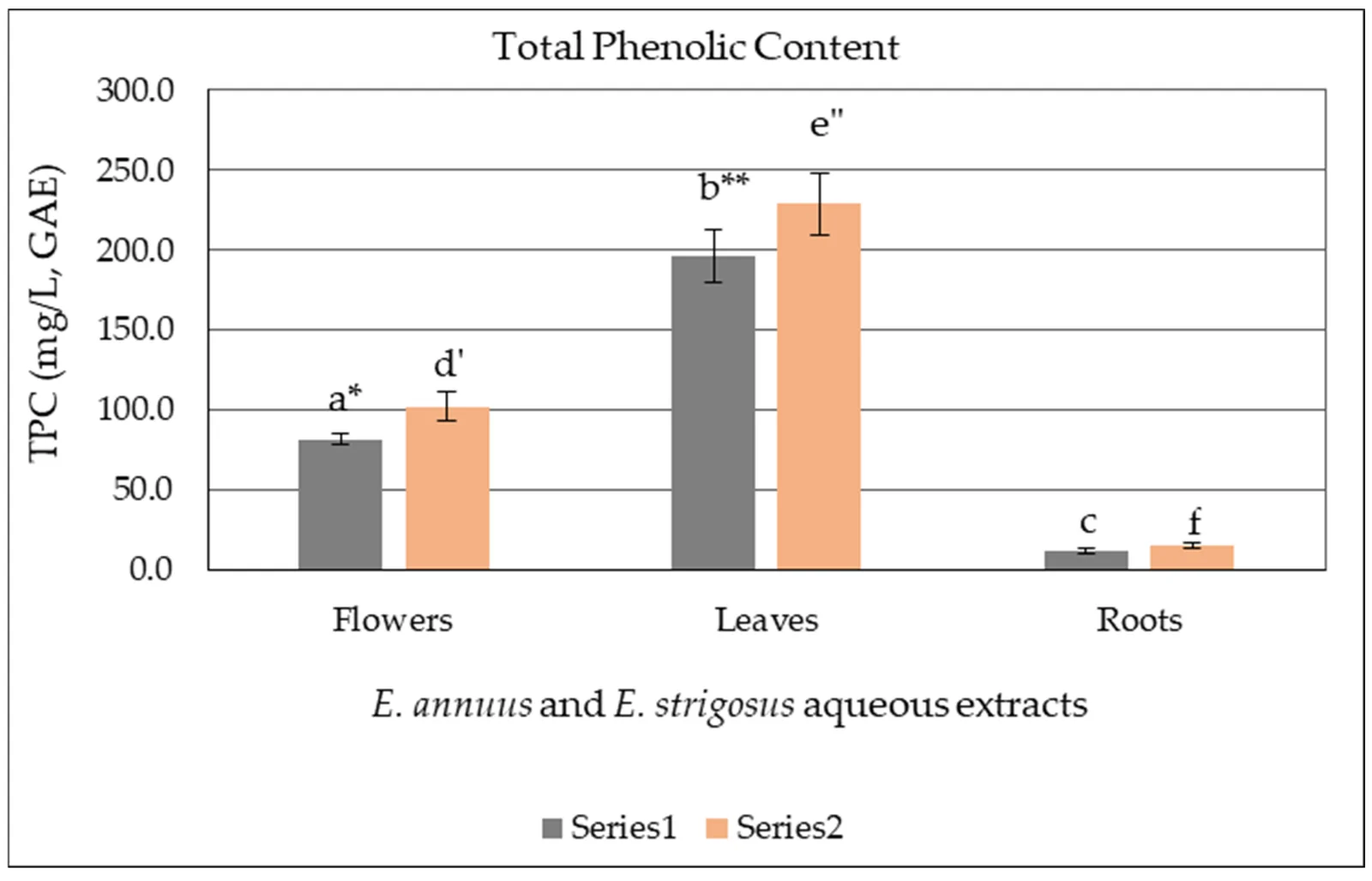
Total phenolic content (TPC, mg/L, expressed in gallic acid equivalent (GAE), *n* = 5) in *E. annuus* (L.) Pers. (Series1) and *E. strigosus* Muhl. ex Willd. (Series2) aqueous extracts (plant material was collected in 2024). Letters (a, b, c and d, e, f) in the columns demonstrate significant differences between TPC values of various plant organs within each species, whereas asterisks (* and **) and apostrophes (′ and ″) indicate significant differences between the same plant organs (flowers and leaves, respectively) of *E. annuus* and *E. strigosus* (*p* ≤ 0.05, using Welch’s *t*-test).

**Figure 4:**
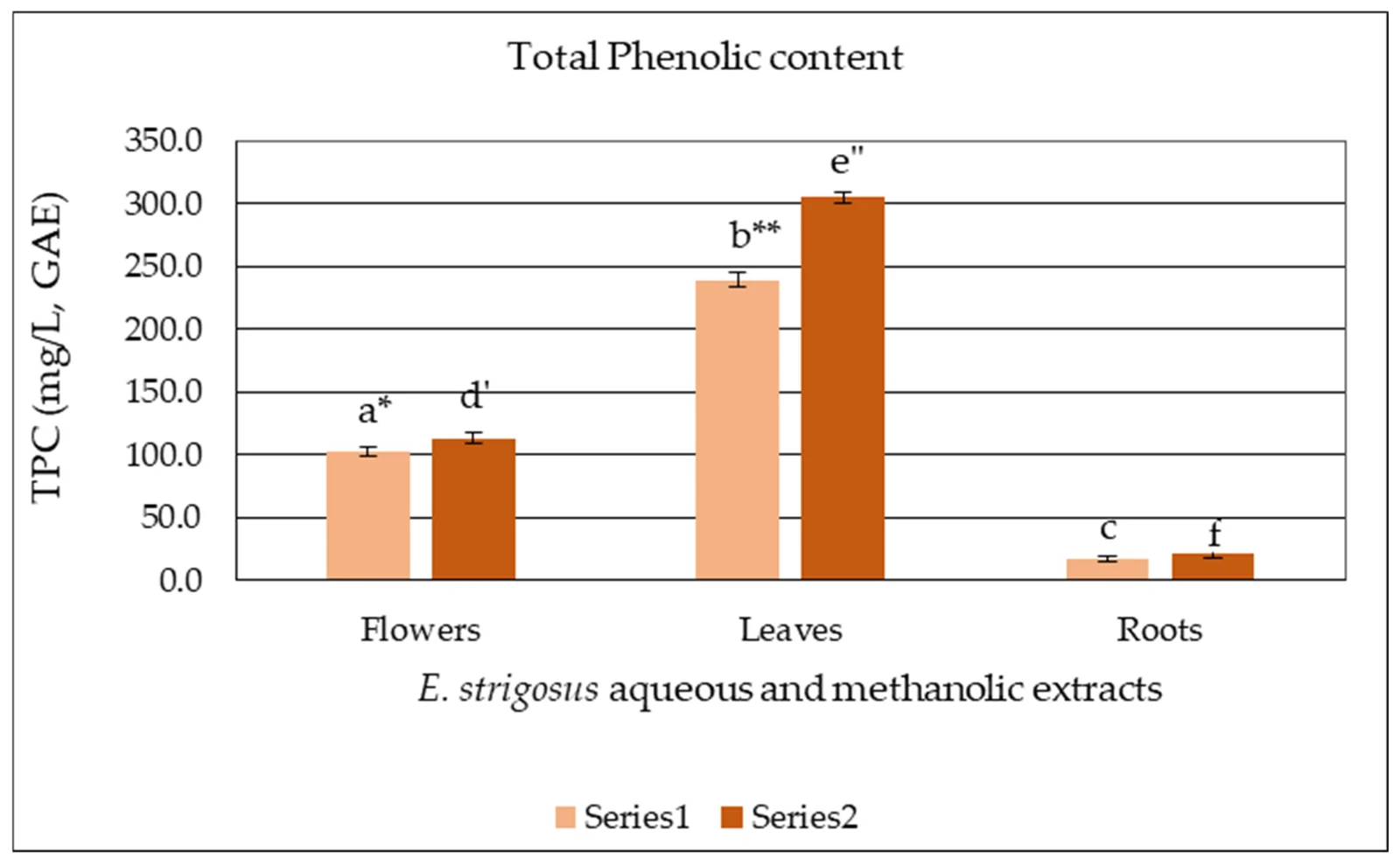
Total phenolic content (TPC, mg/L, expressed in gallic acid equivalent (GAE), *n* = 5) in *E. strigosus* Muhl. ex Willd. aqueous (Series1) and methanolic (1:1, *v*/*v*) (Series2) extracts (plant material was collected in 2025). The letters (a, b, c and d, e, f) in the columns demonstrate significant differences among the TPC values of various plant organs within the species, whereas asterisks (* and **) and apostrophes (′ and ″) indicate significant differences between the aqueous and methanolic extracts of *E. strigosus* (*p* ≤ 0.05, using Welch’s *t*-test).

A significant variation in TPC (determined by the standard Folin–Ciocalteu method, *n* = 5, where *n* indicates the number of measurements performed using the same extract) was observed between the various plant organs in both fleabanes; the order of TPC values was as follows: leaves > flowers > roots (Figure 3 and Figure 4, Tables S1 and S2). However, there are significant differences (*p* ≤ 0.05, using Welch’s *t*-test) between TPC values of aqueous flower and leaf extracts of *E. annuus* and *E. strigosus* (Figure 3, Table S1). The TPC values varied from 11.88 ± 1.27 to 228.79 ± 18.88 (mg/L, GAE, mean ± SD (*n* = 5)) in aqueous *E. annuus* root and *E. strigosus* leaf extracts, respectively (Figure 3, Table S1, herbal material collected in 2024).

It is important to note that the population under investigation of *E. strigosus* Muhl. ex Willd. is confined to a limited area. Consequently, datasets were not generated from a single year’s collection. For this reason, raw material of this fleabane was harvested over two years (2024 and 2025).

For the case of *E. strigosus* plants collected in 2025, the TPC values were found to be higher in methanolic extracts than in aqueous ones, and they varied from 17.17 ± 2.29 to 304.94 ± 3.81 (mg/L, GAE, mean ± SD (*n* = 5)) in aqueous root and methanolic leaf extracts, respectively (Figure 4, Table S2); significant differences were determined among values of water and water/methanol extracts from flowers and leaves.

### 2.5. Antioxidant Ability (AA) of Erigeron annuus (L.) Pers. and Erigeron strigosus Muhl. ex Willd. Extracts

**Figure 5:**
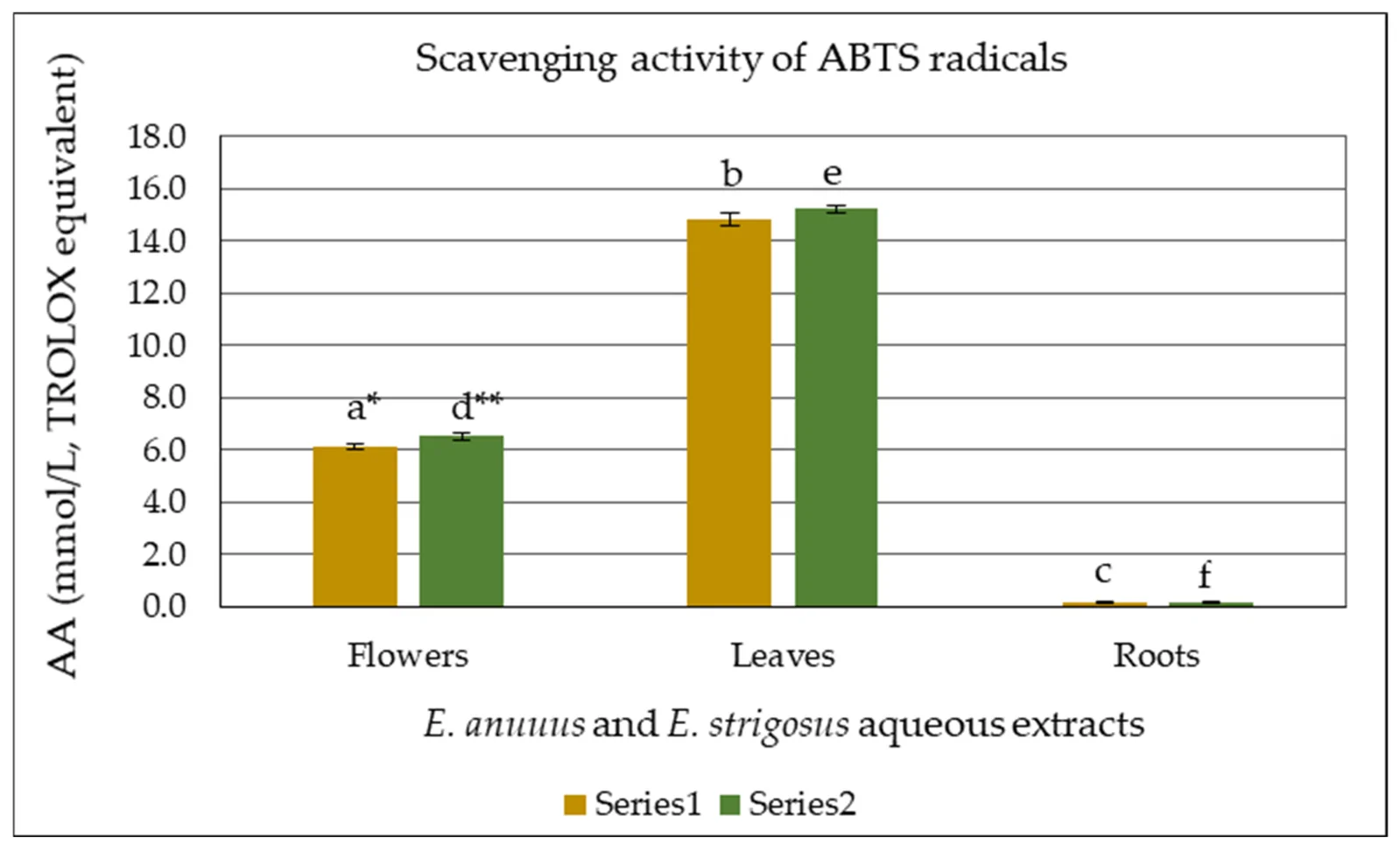
ABTS^●+^ scavenging activity (mmol/L, TROLOX equivalent, *n* = 5) of *E. annuus* (L.) Pers. (Series1) and *E. strigosus* Muhl. ex Willd. (Series2) aqueous extracts. The letters (a, b, c and d, e, f) in the columns indicate significant differences among the ABTS^●+^ scavenging activity values of various plant organs within each species, whereas asterisks (* and **) show significant differences between the aqueous flower extracts of *E. annuus* and *E. strigosus* (*p* ≤ 0.05, using the Welch’s *t*-test).

The free radical-scavenging capacity of both *Erigeron* sp. extracts was determined by the ABTS^●+^ and DPPH^●^ assays. The AA of aqueous *E. annuus* and *E. strigosus* (plant material collected in 2024) extracts, tested by the spectroscopic ABTS^●+^ scavenging method, is presented in Figure 5 and Table S3.

Significant differences were determined between ABTS^●+^ scavenging capacity values of aqueous extracts from various plant organs in each species, and between the two fleabane flower extracts (Figure 5, Table S3).

**Figure 6:**
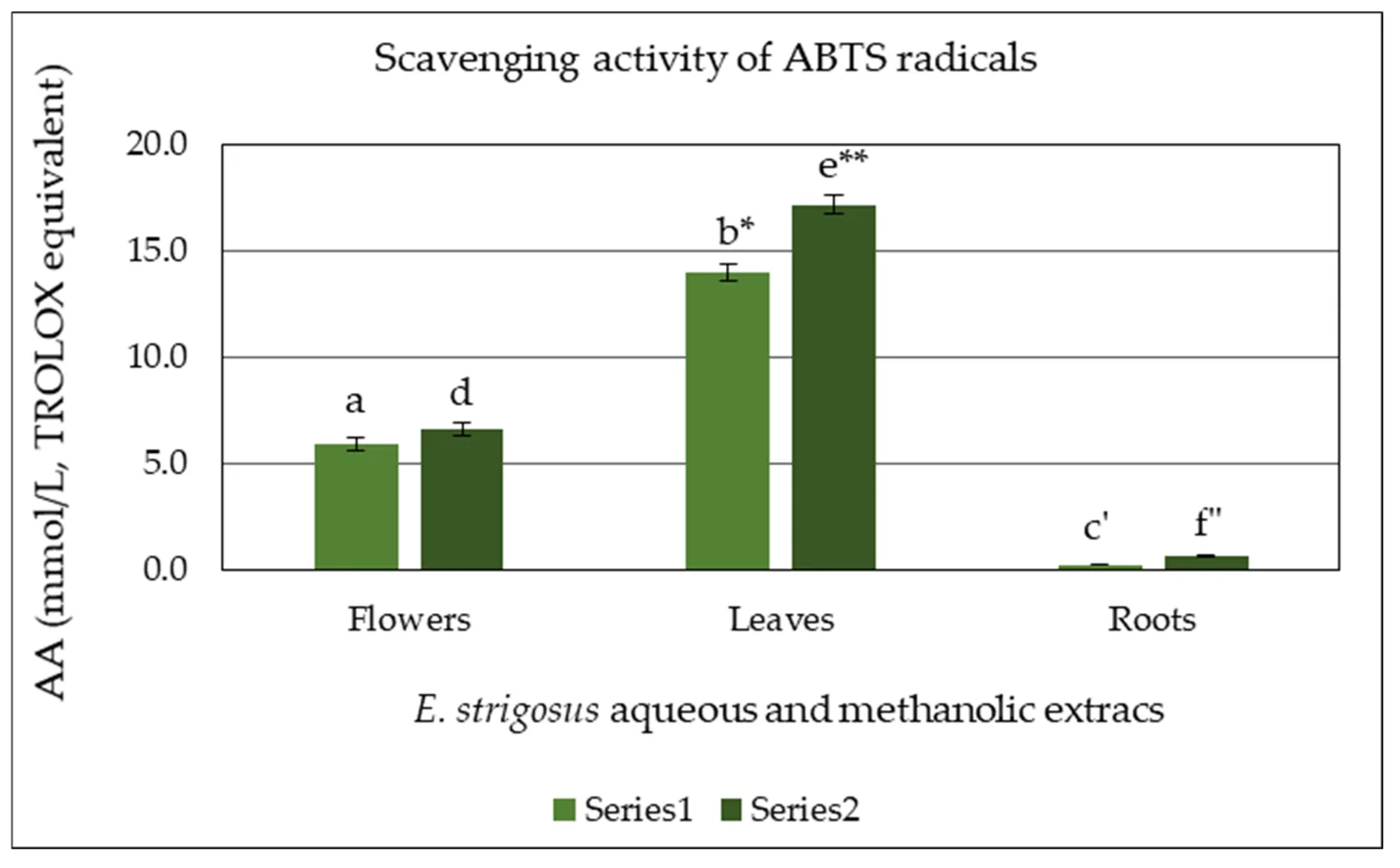
ABTS^●+^ scavenging activity (mmol/L, TROLOX equivalent, *n* = 5) of *E. strigosus* Muhl. ex Willd. aqueous (Series1) and methanolic (1:1, *v*/*v*) (Series2) extracts. The letters (a, b, c and d, e, f) in the columns show significant differences among the ABTS^●+^ scavenging activity values of various plant organs within the species, whereas asterisks (* and **) and apostrophes (′ and ″) indicate significant differences between the aqueous and methanolic *E. strigosus* leaf and root extracts (*p* ≤ 0.05, using the Welch’s *t*-test).

The AA of aqueous and methanolic *E. strigosus* (herbal material collected in 2025) extracts, tested by the spectroscopic ABTS^●+^ scavenging method, is presented in Figure 6 and Table S4.

Significant differences were observed between ABTS^●+^ scavenging capacity values of extracts from various plant organs of *E. strigosus* and between leaf and root extracts in water and in water/methanol (Figure 6, Table S4).

**Figure 7:**
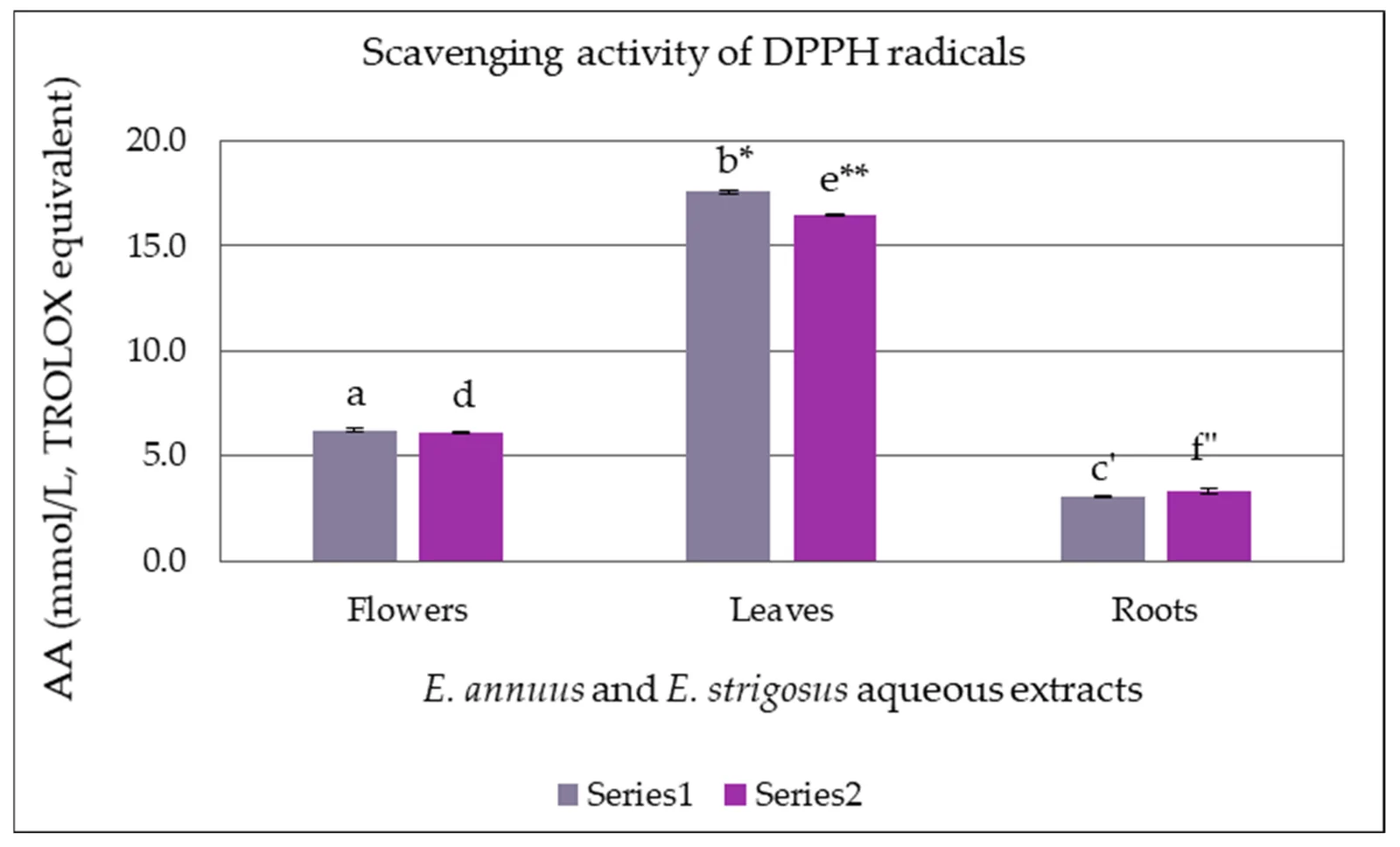
DPPH^●^ scavenging activity (mmol/L, TROLOX equivalent, *n* = 5) of *E. annuus* (L.) Pers. (Series1) and *E. strigosus* Muhl. ex Willd. (Series2) aqueous extracts. The letters (a, b, c and d, e, f) in the columns indicate significant differences among the DPPH^●^ scavenging activity values of various plant organs within each species, whereas asterisks (* and **) and apostrophes (′ and ″) demonstrate significant differences between the aqueous leaf extracts of *E. annuus* and *E. strigosus* (*p* ≤ 0.05, using the Welch’s *t*-test).

The AA of aqueous *E. annuus* and *E. strigosus* (plant material collected in 2024) extracts, tested by the spectroscopic DPPH^●^ scavenging method, is presented in Figure 7 and Table S3.

**Figure 8:**
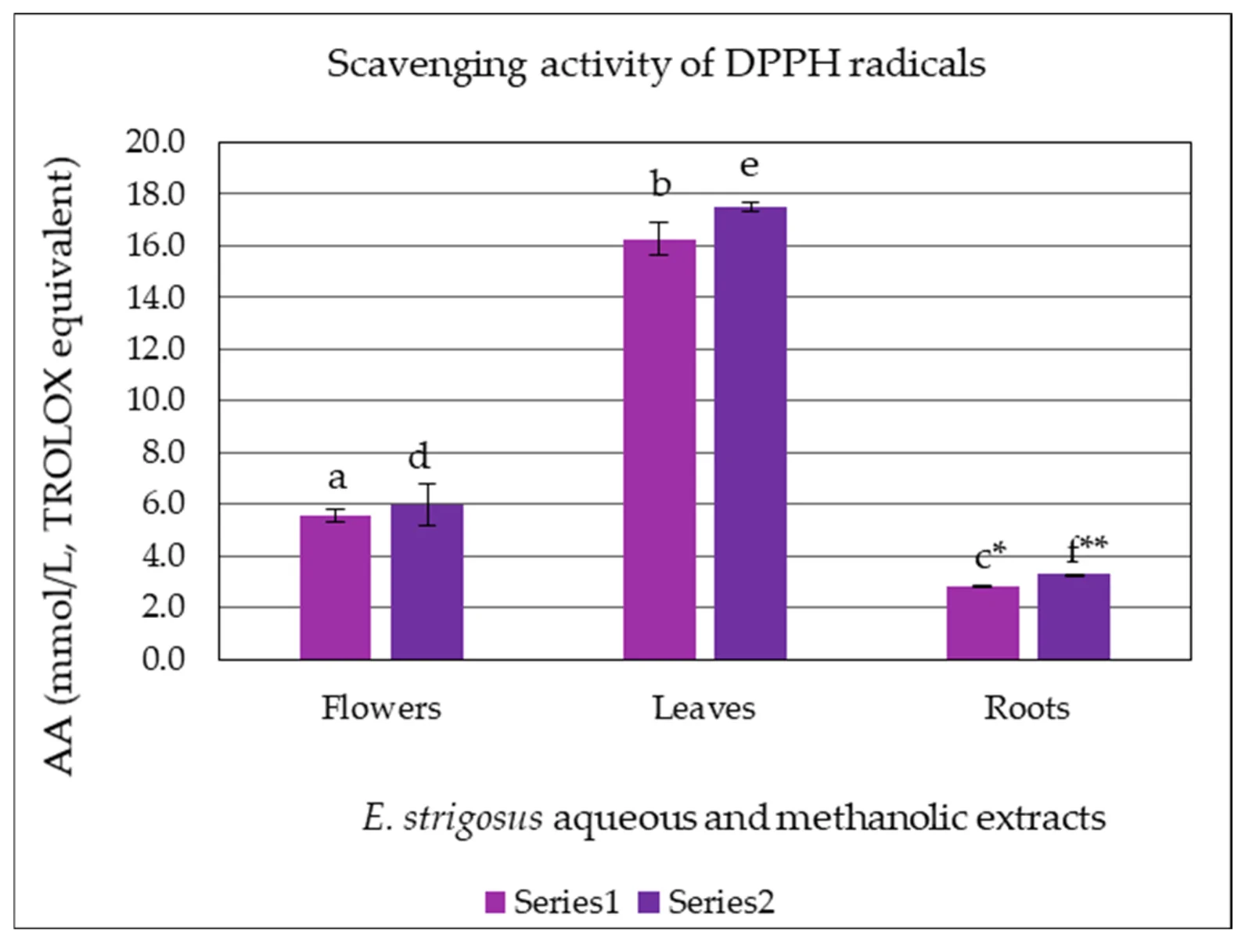
DPPH^●^ scavenging activity (mmol/L, TROLOX equivalent, *n* = 5)) of *E. strigosus* aqueous (Series1) and methanolic (1:1, *v*/*v*) (Series2) extracts. The letters (a, b, c and d, e, f) in the columns demonstrate significant differences among the DPPH● scavenging activity values of various plant organs within the species, whereas asterisks (* and **) indicate significant differences between the aqueous and methanolic root extracts of *E. strigosus* (*p* ≤ 0.05, using the Welch’s *t*-test).

Significant differences were determined in the DPPH^●^ scavenging capacity values of aqueous extracts from various plant organs within each species, as well as between leaf and root extracts of each fleabane species (Figure 7, Table S3). The AA of aqueous and methanolic *E. strigosus* (collected in 2025) extracts, tested by the spectroscopic DPPH^●^ scavenging method, is shown in Figure 8 and Table S4.

Significant differences were revealed between the DPPH^●^ scavenging capacity values of both types of extracts (in water and water/methanol) from *E. strigosus* roots (Figure 8, Table S4).

Pearson correlation analysis of the aqueous extracts of *E. annuus* and *E. strigosus* revealed a significant and strong positive correlation between total phenolic content (TPC) and antioxidant ability (AA) determined by the ABTS^●+^ assay (*E. annus*: r^2^ = 0.999, *p* = 0.01996; *E. strigosus*: r^2^ = 0.9997, *p* = 0.01171), whereas the correlation between TPC and AA, assessed by the DPPH^●^ assay, was also positive (*E. annus*: r^2^ = 0.9845, *p* = 0.1124; *E. strigosus*: r^2^ = 0.9765, *p* = 0.1383), but not statistically significant (*p* > 0.05).

## 3. Discussion

Tentative identification of numerous compounds was achieved in aqueous and methanolic (MeOH:H_2_O, 1:1, *v*/*v*) extracts of *E. annuus* (L.) Pers. and *E. strigosus* Muhl. ex Willd. (Table 1 and Table 2). Some phenolic acids, such as fumaric, succinic, malic, protocatechuic, *p*-coumaric, gelseminic, chlorogenic, quinic, neochlorogenic, isochlorogenic A and B were identified in the extracts of both investigated *Erigeron* species. Additionally, umbelliferone, loliolide, kaempferol/luteolin, erigeside I, baicalin, quercetin and its glucoside, apigenin and its glucuronide, apigenin-7-O-glucuronide-6′-ethyl ester, astragalin/quercitrin, scutellarin, caffeoyl shikimic acid glucoside, sesquiterpene hydrocarbon(s) and rutin were found to be the common compounds in all investigated fleabane extracts.

Most of the acids, such as succinic, quinic, betulinic, ursolonic, protocatechuic, *p*-coumaric, chlorogenic, and isochlorogenic acid A and B, found in annual fleabane extracts under study were also reported previously in *E. annuus* extracts [18,20,23,24,27,28].

A list of the same compounds identified by us as those reported in the literature reports of *E. annuusis* is as follows: coumarin, umbelliferone [18]; flavonoids, kaempferol [18,33], quercetin [18,33], apigenin [17,21,22,39], apigenin-7-*O*-glucuronide [17,21], astragalin [21], erigeside I [21,29,39], quercetin 3′-*O*-glucoside [22,33], hispidulin [29], erigeroflavanone [30,31], luteolin [27,33], apigenin-7-*O*-glucuronide-6′-ethyl ester [39]; and lactone loliolide [29] (Table 1). It should be mentioned that caffeic acid [19,20,22,27] and pyromeconic acid [21,23,26,29], frequently determined by other researchers in *E. annuus* extracts, were not found by us (or their quantities were below detection limits) in annual fleabane extracts (Table 1). It is well known that *γ*-pyranone derivatives are characteristic of *E. anuuus* [12,39]. We identified arenol and arzanol (Table 1), α-pyrone derivatives, which are common constituents of *Helichrysum* sp. [53,54].

There is very limited data in the literature regarding the phytochemistry of *E. strigosus* extracts [47]. Compounds such as oleanolic [18] and pyromeconic [17,21,23,26,29,47] acid, 5,7-dihydroxychromone [29], catechin [18], (-)-syringaresinol-4-*O*-*β*-D-glucopyranoside [39], and apigenin 7-*O*-methylglucuronide [32] documented previously in the reports of *E. annuus* were determined in our *E. strigosus* extracts (Table 2). Scutellarin, baicalin, and baicalein, identified in both fleabane extracts, are known as characteristic phytochemicals in *Scutellaria* sp. [55,56,57]. However, matricaria lactone, lachnophyllum ester and sesquiterpene hydrocarbons (Table 1 and Table 2) have been identified earlier in *Erigeron* species [12,13,14,15,16].

To the best of our knowledge, a number of documented reports related to the in vivo and/or in vitro toxic capacity of both *Erigeron* species (*E. anuuus* and *E. strigosus*) are very limited. The cytotoxicity tests performed by Thakur et al. (using 3-(4,5-dimethylthiazol-2-yl)-2,5-diphenyltetrazolium bromide (MTT) assay) revealed that 50% viability of the HEK293 cell was observed at a 50 μg/mL concentration of ethyl acetate fraction for the annual fleabane extract [34]. Additionally, investigations into the in vitro phytotoxic activity of *E. anuuus* were reported [23,46]. The germination inhibitory potential of methanolic extracts from *E. annuus* flowers on lettuce seeds was tested by Oh et al., and the results showed that the ethyl acetate-soluble fraction had the most significant inhibitory effects (at the 6000 ppm concentration) [23]. Liu et al. [46] examined the effects of water extracts from *Erigeron canadensis* L. and *Erigeron annuus* (L.) Desf. plants at various concentrations on the germination and seedling growth of three major food crops, including wheat (*Triticum aestivum* L.), rice (*Oryza sativa* L.), and corn (*Zea mays* L.), using the Petri dish method. Results obtained in the above study showed that *E. annuus* extracts at a 100 g/L concentration completely suppressed the germination of wheat and rice [46]. The data obtained in the study on the in vivo toxicity of aqueous *E. anuuus* and *E. strigosus* extracts (dosages ranging from 100 μL to 4.5 mL), using brine shrimp (*Artemia* sp.) larvae tests, are of significant importance and have filled a considerable gap in this area of research. It was revealed that the extracts from the leaves of both *Erigeron* species were the most toxic when compared to other organs (flowers and roots) (Table 3). The toxicity of rough fleabane flower and root extracts was found to be slightly higher than that of annual fleabane corresponding extracts.

Another biological property that can be used to evaluate the pharmacological potential of plants is antioxidant ability (AA). AA test results, using spectrophotometric radical (ABTS^●+^ and DPPH^●^) scavenging assays, are presented in Figure 5, Figure 6, Figure 7 and Figure 8 (Tables S3 and S4). The majority of the phenolic constituents (predominantly acids and flavonoids), identified in the extracts in this study (Table 1 and Table 2), have the capacity to demonstrate different levels of antioxidant potential. Typically, AA tests are accompanied by TPC, and a positive correlation is frequently observed between these two variables.

Many scientific studies have improved the method used to prepare extracts, the characteristics of the herbal material, the type of solvents or their mixtures, and the temperature and duration of the extraction procedure, etc., all of which strongly influence the qualitative as well as quantitative extraction of bioactive compounds from herbal material [19,22,24,26,34,58,59,60]. However, the findings of different antioxidant tests in the existing literature with regard to *E. annuus* [12,19,22,24,25,26,34,41,42] are not always commensurable, owing to the varying methodologies and formats in which these data are presented. In the current research, aqueous leaf *E. annuus* extracts (prepared in ultrasound for 30 min at room temperature) containing 196.92 ± 16.69 (mg/L, GAE) of TPC (Figure 3, Table S1) showed the highest values 14.83 ± 0.25 and 17.55 ± 0.07 (mmol/L, TROLOX equivalent) of ABTS and DPPH radical-scavenging potential, respectively (Figure 5 and Figure 7, Table S3). It is evident that a comparison of the results obtained with those of very different data in the literature is complicated. For example, TPC in the butanol fraction, prepared from crude *E. annuus* leaf extract (which was made at 70 °C in methanol for 2 h), was the highest (396.49 mg of GAE/g), followed by the water and chloroform fractions (241.87 and 107.34 mg of GAE/g, respectively) [19]. Moreover, this fraction exhibited the highest antioxidant effects in the ABTS radical-scavenging and ferric-reducing antioxidant power (FRAP) assays in the above study. In other studies, the fractions of the crude extract of *E. annuus* in methanol and butanol showed significant scavenging effects on DPPH^●^ and peroxynitrite [22]. Extracts of whole annual fleabane, prepared in ethanol/water (50:50, *v*/*v* for 2 h at room temperature three times) containing an average of 187.12  mg/g (of residue) of TPC, exhibited IC_50_ (μg/mL) values of 125 ± 1.06 and 146 ± 14.3 for ABTS decolorization and DPPH scavenging, respectively [24]. Zhang et al. [26] determined that TPC in *E. annuus* flowers using different solvents (extraction with heating for 6 h) varied from 31.6 to 152.8 mg (GAE/g dry extract) and the aqueous extracts exhibited the highest TPC values (an average of 152.8 mg/g), followed by acetone, chloroform, ethanol and methanol (115.5, 103.7, 92.6 and 89.9 mg/g, respectively); by contrast, the methanol extract showed the strongest ability to scavenge DPPH radicals [26]. In other study, it was revealed that the ethyl acetate fraction of the annual fleabane crude extract (prepared by methanol in an orbital shaker at 40 °C for 72 h) containing the highest TPC (77.29 ± 0.11 mg/g, expressed in GAE mg/g of crude extract and dry weight of fraction) demonstrated the strongest DPPH radical-scavenging ability (46.71 ± 0.67 μg/mL) and FRAP effects; there is a high potential of polyphenols to reduce α-amylase activity [34].

It should be mentioned that there is a lack of available data related to *E. strigosus*. In the present research, aqueous extracts of rough fleabane leaves, containing 228.79 ± 18.88 (mg/L, GAE) of TPC (Figure 3, Table S1), exhibited the highest values of 15.22 ± 0.13 and 16.45 ± 0.04 (mmol/L, TROLOX equivalent) for ABTS and DPPH radical-scavenging activities, respectively (Figure 5 and Figure 7, Table S3). Additionally, it was revealed that methanolic extracts of *E. strigosus* leaves, containing 304.94 ± 3.81 (mg/L, GAE) of TPC (Figure 4, Table S2), exhibited the strongest ABTS^●+^ and DPPH^●^ scavenging potential (17.15 ± 0.43 and 17.50 ± 0.20 (mmol/L, TROLOX equivalent), respectively) (Figure 6 and Figure 8, Table S4). For the first time, toxicity, TPC, ABTS and DPPH radical-scavenging ability have been determined for *E. strigosus* extracts, and the latter results cannot be compared because there is no data already published on this subject.

## 4. Materials and Methods

### 4.1. Plant Material

The raw plant material of *E. annuus* (L.) Pers. and *E. strigosus* Muhl. ex Willd. (fresh weight up to 1 kg) was collected in the full blooming stage (at the end of July 2024 and 2025) in Lithuania (Alytus County, Lazdijai district, Šlavantai and Kaunas County, Prienai district, Būdvietis). Harvesting locations are indicated in Figure S1. The population of *E. strigosus* Muhl. ex Willd. is limited in this area. To avoid reducing or even destroying the population, it was necessary to harvest as little as possible of the raw material. For these reasons, we did not have enough plant material for the experiments from a single year’s harvest; therefore, it was necessary to collect it over two years (2024 and 2025). The entire plant of fleabane was gathered in a randomized manner, and afterward three morphological organs, including the roots, leaves, and inflorescences, were separated prior to drying. The collection and identification of the herbal material of *E. strigosus* Muhl. ex Willd. and *E. annuus* (L.) Pers. was performed under supervision of plant taxonomist Dr. Z. Gudžinskas (Laboratory of Flora and Geobotany, Nature Research Centre, Vilnius, Lithuania). Images of both *Erigeron* sp. plants are presented in Figure S2. The plant material was transferred to the laboratory as soon as possible for drying at room temperature (20–25 °C) in the shade with ventilation for 3 weeks (until a constant moisture content was achieved). A traceable humidity metre (8709 Pen type hydrometer, Fisher Scientific, Webster, TX, USA) was used to measure the humidity level of herbal material. The whole herbal material was stored in tightly closed paper bags at a temperature of 20 ± 2 °C and a low level of humidity until analysis (up to 6 months).

### 4.2. Preparation of Extracts for HPLC/DAD/TOF Analysis, Toxicity Tests and TPC Determination

Samples of air-dried inflorescences and the leaves and roots of *E. strigosus* and *E. annuus* were ground into a homogeneous powder. Extracts were made from 2.5 g of crushed herbal material and 25 mL of distilled water (ratio of herbal material to water, 1:10 *w*/*w*); the extraction duration was 30 min at room temperature (23.0 ± 3.0 °C). For toxicity tests, the ratio 1:20 of dried herbal material to water was used at a concentration of 0.05 g/L (dry plant material). Afterward, the mixtures were filtered through filter paper (11 μm pore size filter paper (Whatman) and then through nylon syringe filters (0.22 mm) before chromatographic analysis. Additionally, 50% methanolic extracts were prepared by the same procedure, using a mixture of methanol and water (1:1, *v*/*v*).

### 4.3. HPLC-DAD-MS (TOF) Analysis

Chemical composition of aqueous and methanolic extracts of *Erigeron* sp. (*E. annuus* and *E. strigosus*) inflorescences, leaves, and roots was identified using HPLC (High-Performance Liquid Chromatography)/DAD (Diode Array Detector)/TOF (Time of Flight Mass Spectrometer) (Agilent 1260 Infinity (Agilent Technologies, Waldbronn, Germany) and the Agilent 6224 TOF (Agilent Technologies, Santa Clara, CA, USA)) technique. A column Hypersil GOLD^TM^ (particle size of 1.9 µm; column parameters of 150 × 2.1 mm) (Thermo Scientific, Vilnius, Lithuania) was applied. Column temperature was set to 40 °C during the analysis. The gradient system was applied as follows: A (deionized water, containing 0.1% formic acid) and B (acetonitrile, containing 0.1% formic acid). Chromatographic separation was performed at a flow rate of 0.3 mL/min in the HPLC system using the following stepwise gradient elution method presented in Table S5. The ionization interface (ESI) provided ionization in positive and negative modes. Samples were injected by an autosampler at a volume of 4 to 8 µL. DAD spectra were recorded in a range from 210 to 400 nm. MS (TOF) acquisition parameters were identical to those in our previous research [61]. Acetonitrile and formic acid were purchased from Honeywell (Seelze, Hanover, Germany). Standards of main phenolic acids (fumaric, succinic, caffeic, syringic, gallic, chlorogenic, etc., with purity ≥ 95–99%) and flavonoids, including quercetin-3-*O*-glucoside and apigenin-7-*O*-glucoside, were purchased from Merck and Sigma-Aldrich Solutions (Darmstadt, Germany). References of flavanols, (-)-epicatechin and (+)-catechin hydrate were bought from Fisher Scientific (Loughborough, Leicestershire, UK), and rutin (97+%) was received from Acros Organics (Geel, Belgium). Baicalin (98% purity) (glycoside flavonoid) and baicalein (5,6,7-trihydroxyflavone) (flavone) were obtained from Lonza Bioscience (Walkersville, MD, USA). Apigenin and quercitrin dihydrate were purchased from Fluka AG (Buchs, SG, Switzerland). Total ion chromatograms of *E. annuus* and *E. strigosus* leaf extracts in both positive and negative ionization modes are included in Figures S3 and S4, respectively.

### 4.4. Toxicity Tests

Toxic activity was tested in vivo, using brine shrimp *Artemia salina* (larvae) [62]. The eggs of shrimps hatched within 48 h to provide larvae (nauplii) in seawater (32 g/L NaCl in water) at 20–25 °C; the number of shrimps varied from 11 to 16 in a 5 mL volume. For the toxicity assay, different volumes of *E. strigosus* and *E. annuus* aqueous extracts were used: 100 and 500 μL, and 1–4.5 mL (2, 3 and 4.5 mL of extracts also contained ≈2.5% NaCl). The salinity employed in the assay was maintained at a level that was found to be compatible with the hatching and survival conditions for *A. salina*. Control tests were performed in salt water. Survivors were counted after 24 h. At least three repetitions were carried out for each different extract amount.

### 4.5. Spectrophotometric ABTS^●+^ and DPPH^●^ Scavenging Assays

The AA of aqueous *E. strigosus* and *E. annuus* extracts was determined by the spectroscopic methods described in detail in the literature [63]. The calibration curve for TPC determination (in a range from 0 to 500 mg/L GAE) is presented in Figure S5. Calibration curves for AA determination by ABTS^●+^ and DPPH^●^ scavenging assays (with a range up to 200 m–mol/L TROLOX equivalent) are shown in Figures S6 and S7, respectively.

### 4.6. Statistical Data Analysis

All data obtained during the study were statistically analyzed using the Analyse-it add-in for Microsoft Excel (free trial version). The results are presented as an average value with standard deviation (SD). The correlation coefficient r between TPC and free radical-scavenging capacity was calculated using Pearson’s correlation method. Significant differences between samples were calculated using a two-sample *t*-test (Welch’s *t*-test), and *p*-values less than 0.05 were considered significantly different.

## 5. Conclusions

The present research contributes to our knowledge of the phytochemistry, toxicity and biological properties (including antioxidant activity) of two species of *Erigeron* plants, *E. annuus* (L.) Pers. and *E. strigosus* Muhl. ex Willd., collected from the wild flora. As in numerous countries worldwide, both species are considered alien to Lithuania, and even more so, *E. annuus* is regarded as an invasive, aggressive weed. An intraspecific diversity of *Erigeron* species was revealed in rather limited growing areas. To the best of our knowledge, until now, research data regarding this subject have been extremely limited.

A list of various compounds, such as phenolic acids, lactones, coumarins, pyrone derivatives and flavonoids, present in *E. annuus* and *E. strigosus* extracts was documented in the present research. Our study has reduced the shortage in the phytochemistry of *E. annuus,* with a particular focus on *E. strigosus* plants. To the best of our knowledge, up to now, there are very limited data regarding the phytochemistry and bioactivity of *E. strigosus* extracts.

Data obtained during the study on the toxicity of *E. annuus* and *E. strigosus* aqueous extracts, using brine shrimp *(Artemia* sp.) larvae tests, is of great importance, filling a gap in the research related to the toxicity of *Erigeron* species. TPC was found to be 11.88 ± 1.27–304.94 ± 3.81 (mg/L, GAE) in aqueous *E. annuus* roots and methanolic *E. strigosus* leaf extracts, respectively. A positive correlation was observed between TPC and ability of the extracts to scavenge free radicals. Average values of ABTS^●+^ and DPPH^●^ scavenging capacity were determined to be 0.17–14.83 and 3.06–17.55 (mmol/L TROLOX equivalent) for *E. annuus* aqueous extracts from different plant organs, respectively. In the case of *E. strigosus* aqueous and methanolic extracts from different plant organs, ABTS radical-scavenging activity ranged from 0.16 ± 0.02 to 17.15 ± 0.43, and DPPH^●^ scavenging ability varied from 2.83 ± 0.01 to 17.50 ± 0.20 (mmol/L, TROLOX equivalent). The order of toxicity, TPC and antioxidant ability for the various fleabane plant organs was found as follows: leaves > flowers > roots. Significant differences were observed between the values. A strong positive correlation between TPC and ABTS^●+^ scavenging capacity was revealed, while the correlation between TPC and DPPH^●^ assay values was also positive, but did not reach statistical significance (*p* > 0.05).

This paper elucidates the gaps in research related to the phytochemistry and bioactivity of *Erigeron* species, thus contributing significantly to the advancement of knowledge in this field. Moreover, the study revealed the importance of investigating phytochemistry to improve our understanding of its role in the spread of alien and invasive species. Further investigations on primary and secondary metabolites of wild-growing *Erigeron* sp. for pharmacological purposes are promising.

## Figures and Tables

**Table 1:** Tentative identification of compounds in inflorescence, leaf and root extracts of *Erigeron annuus* (L.) Pers. analyzed by HPLC-DAD-TOF technique.

Constituents	Formula	Molecular Weight	*m*/*z* ESI^+^ (Da)	*m*/*z* ESI^−^ (Da)	UV Absorb., λ_max_, nm	Literature **
Acids:						
* Fumaric ^L^	C_4_H_4_O_4_	116.1	116.072		210	
* Succinic ^L^	C_4_H_6_O_4_	118.1	118.084		220	[24]
Glutaric ^F^	C_5_H_8_O_4_	132.1	133.061		210	
Malic ^R^	C_4_H_6_O_5_	134.1		133.018	340	
Protocatechuic ^R^	C_7_H_6_O_4_	154.1	155.975		260; 290	[20]
*p*-Coumaric ^F^	C_9_H_8_O_3_	164.2	166.086		260	[23]
Quinic acid ^F, L, R^	C_7_H_12_O_6_	192.2		191.017	195	[27]
Gelseminic (Scopoletin) ^L^	C_10_H_8_O_4_	192.2		190.986	340	
* Chlorogenic (3-*O*-caffeoylquinic) ^L, F, R^	C_16_H_18_O_9_	354.3	355.106	353.092	210; 315	[27,28]
Neochlorogenic (5-*O*-caffeoylquinic) ^L, F, R^	C_16_H_18_O_9_	354.3	355.097	353.089	210; 315	
Betulinic/Ursolonic ^L^	C_30_H_48_O_3_	456.7		456.885	210	[18]
Isochlorogenic (3,5-Dicaffeoylquinic) acid A ^L, F^	C_25_H_24_O_12_	516.5	517.134	515.123	325	[20,21,27]
Isochlorogenic (3,4-Dicaffeoylquinic) acid B ^R^	C_25_H_24_O_12_	516.5	517.134	515.148	325	[21,27]
Other:						
Umbelliferone ^F, L^	C_9_H_6_O_3_	162.1	163.039	160.981	300	[18]
Matricaria lactone ^F, L^	C_10_H_10_O_2_	162.2	163.039	160.981		[15,16]
Loliolide ^F, L, R^	C_11_H_16_O_3_	196.2		195.055		[29]
Isoscutellarein ^F, L^	C_15_H_10_O_6_	286.2		285.043	275	[12]
Kaempferol/Luteolin ^F^	C_15_H_10_O_6_	286.2		285.046	265; 360	[18,27,33]
Sesquiterpene hydrocarbon(s) ^F, L^	C_15_H_24_	204.4	205.096			[12,14,15,16]
Apigenin ^F^	C_15_H_10_O_5_	270.2		269.048	210; 325	[17,21,22,39]
Acacetin (Linarigenin; 5,7-Dihydroxy-4′-methoxyflavone) ^F^	C_16_H_12_O_5_	284.3		282.942		
Hispidulin (Scutellarein 6-methyl ether; 4′,5,7-Trihydroxy-6-methoxyflavone) ^L^	C_16_H_12_O_6_	300.3		298.918		[29]
* Quercetin ^F^	C_15_H_10_O_7_	302.2		301.038	225; 255; 370	[18,32]
Arenol ^F^	C_21_H_24_O_7_	388.4		386.926		
Arzanol ^F, L^	C_22_H_26_O_7_	402.4		401.026		
Erigeroflavanone ^F, L^	C_19_H_18_O_10_	406.3	407.033	405.031		[30,31]
Erigeside I (6′-*O*-Caffeoylerigeroside) ^F, L^	C_20_H_20_O_11_	436.4		435.008		[21,29,39]
* Baicalin (7-D-Glucuronic acid-5,6-dihydroxyflavone) ^F, L^	C_21_H_18_O_11_	446.4	447.099	444.968	275; 315	
Apigenin-7-*O*-glucuronide ^F, L, R^	C_21_H_18_O_11_	446.4	447.099	445.083		[17,21]
Astragalin (Kaempferol 3-*O*-glucoside)/Quercitrin (Quercetin 3-rhamnoside) ^F^	C_21_H_20_O_11_	448.4		446.956	265; 346	[21]
Scutellarin (Scutellarein-7-*O*-glucuronide; Breviscapin) ^F, L, R^	C_21_H_18_O_12_	462.4	463.084	461.078	285, 335	
Quercetin 3′-*O*-glucoside ^R^	C_21_H_20_O_12_	464.4	465.104	463.084	255; 365	[22,33]
Apigenin-7-*O*-glucuronide-6′-ethyl ester ^L, R^	C_23_H_22_O_11_	474.4	475.084			[39]
Isorhamnetin 3-*O*-glucoside ^F^	C_22_H_22_O_12_	478.4		476.954		
Caffeoylshikimic acid glucoside ^F, L^	C_22_H_26_O_13_	499.1	499.12			
Rutin ^F^	C_27_H_30_O_16_	610.5		609.130	255; 355	

* Additional identification with a reference compound. ^F, L, R^ indicate inflorescence (F), leaf (L) and root (R) extracts, respectively. ** Previously reported in the literature related to *Erigeron annuus* (L.).

**Table 2:** Tentative identification of main compounds in *Erigeron strigosus* Muhl. ex Willd. inflorescence, leaf and root extracts analyzed by HPLC-DAD-TOF technique.

Compounds	Formula	Molar Mass	*m*/*z* ESI^+^, (Da)	*m*/*z* ESI^−^ (Da)	UV Absorb., λ_max_, nm
Acids:					
Pyromeconic (3-hydroxy-pyran-4-one) ^F^	C_5_H_4_O_3_	112.1	113.022		210; 270
* Fumaric ^F^	C_4_H_4_O_4_	116.1	116.072		210
* Succinic ^F^	C_4_H_6_O_4_	118.1	118.084		220
Malic ^F, L, R^	C_4_H_6_O_5_	134.1		133.018	340
Protocatechuic ^F^	C_7_H_6_O_4_	154.1	156.042		260; 295
*p*-Coumaric ^F, L, R^	C_9_H_8_O_3_	164.2	166.040	163.081	260
* Caffeic acid ^F^	C_9_H_8_O_4_	180.2	181.122		220; 295–325
Gelseminic (scopoletin) ^F, L^	C_10_H_8_O_4_	192.2		191.061	340
Quinic ^F, R^	C_7_H_12_O_6_	192.2		191.108	195
* Syringic ^R^	C_9_H_10_O_5_	198.2	199.061		210; 260
Linolenic (9,12,15-octadecatrienoic) ^F, L, R^	C_18_H_30_O_2_	278.5	279.157	277.225	210
9,12,13-Trixydroxy-10*E*-octadecenoic ^F, R^	C_18_H_34_O_5_	330.5	331.189	329.238	
* Chlorogenic (3-*O*-caffeoylquinic) ^F, L, R^	C_16_H_18_O_9_	354.3	355.108	353.092	210; 315
Neochlorogenic (5-*O*-caffeoylquinic) ^L, F, R^	C_16_H_18_O_9_	354.3	355.097	353.089	210; 315
3-*O*-Feruloylquinic ^L, R^	C_17_H_20_O_9_	368.3		367.145	
Oleanolic ^R^	C_30_H_48_O_3_	456.7		455.236	195; 230
Isochlorogenic (3,5-dicaffeoylquinic) acid A ^F, L, R^	C_25_H_24_O_12_	516.5	517.155	515.148	325
Isochlorogenic (3,4-dicaffeoylquinic) acid B ^F^	C_25_H_24_O_12_	516.5	517.134	515.124	325
Other:					
Coumaran (2,3-dihydrobenzofuran) ^F, L, R^	C_8_H_8_O	120.2	121.052		210
Isoamyl butyrate ^L, R^	C_9_H_18_O_2_	158.2	158.996		210
Umbelliferone ^L, R^	C_9_H_6_O_3_	162.1	163.039		300
Lachnophyllum ester ^F, L^	C_11_H_12_O_2_	176.1	177.092	175.10	
5,7-Dihydroxychromone ^L^	C_9_H_6_O_4_	178.1	179.108	177.025	
Loliolide ^F, L^	C_11_H_16_O_3_	196.2	197.118	195.055	
Sesquiterpene hydrocarbon(s) ^L^	C_15_H_24_	204.4	205.096		
* Baicalein ^F, L^	C_15_H_10_O_5_	270.2	271.228	269.051	270; 330
Apigenin ^F, L^	C_15_H_10_O_5_	270.2	271.228	269.051	210; 230; 325
Kaempferol/Luteolin ^L^	C_15_H_10_O_6_	286.2		285.046	265; 364
* Epicatechin ^F, L, R^	C_15_H_14_O_6_	290.3	291.098		280
* Catechin ^F, R^	C_15_H_14_O_6_	290.3	291.233		280
* Quercetin ^F^	C_15_H_10_O_7_	302.2	303.233	301.040	225; 255; 370
Quercetin 3′,4′,7-trimethyl ether ^F, L^	C_18_H_16_O_7_	344.3	346.259	343.042	
Erigeside I (6′-*O*-Caffeoylerigeroside) ^F, L, R^	C_20_H_20_O_11_	436.4	437.104	435.096	
Erigerol ^L^	C_25_H_40_O_6_	436.6		435.094	
* Baicalin ^F, L^	C_21_H_18_O_11_	446.4	447.099	445.083	275; 315
Apigenin-7-*O*-glucuronide ^F, L^	C_21_H_18_O_11_	446.4	447.099	445.083	
Astragalin/Quercitrin ^L^	C_21_H_20_O_11_	448.4		447.229	265; 345
(-)-Epigallocatechin gallate ^R^	C_22_H_18_O_11_	458.4		457.251	275
Apigenin 7-*O*-methylglucuronide ^F, R^	C_22_H_20_O_11_	460.4	461.281	459.148	
Scutellarin (scutellarein-7-*O*-glucuronide) ^F, L^	C_21_H_18_O_12_	462.4	463.085		285; 335
* Quercetin 3′-*O*-glucoside ^F, L^	C_21_H_20_O_12_	464.4	465.104	463.094	255; 365
Apigenin-7-*O*-glucuronide-6′-ethyl ester ^F, L^	C_23_H_22_O_11_	474.4	475.085		
Caffeoylshikimic acid glucoside ^L,^	C_22_H_26_O_13_	498.5	499.124		
(-)-Syringaresinol-4-*O*-*β*-D-glucopyranoside ^F, R^	C_28_H_36_O_13_	580.6	582.145	579.182	
* Rutin ^R^	C_27_H_30_O_16_	610.5	611.426		255; 355

^L, F, R^ Presence of compounds in leaf (L), inflorescence (F) and root (R) extracts. * Additional identification using compound standards.

**Table 3:** Brine shrimp *Artemia salina* nauplii lethality (average ± SD (standard deviation), *n* = 3, *n* refers to the number of measurements taken at the same extract dose) in flower, leaf and root extracts of *E. annuus* (L.) Pers. and *E. strigosus* Muhl. ex Willd.

	*Artemia salina* Nauplii Mortality, %					
Dosage *	*E. annuus*			*E. strigosus*		
Volume, mL	Flowers	Leaves	Roots	Flowers	Leaves	Roots
0.1	0.0 ± 0.0	6.1 ± 8.6	0.0 ± 0.0	0.0 ± 0.0	0.0 ± 0.0	0.0 ± 0.0
0.5	34.8 ± 10.7	72.3 ± 2.4	0.0 ± 0.0	47.2 ± 5.0	66.7 ± 4.3	0.0 ± 0.0
1.0	38.6 ± 3.9	82.8 ± 1.3	4.8 ± 6.7	87.7 ± 9.0	97.9 ± 2.9	15.2 ± 4.3
2.0	80.1 ± 5.4	100 ± 0.0	51.5 ± 5.1	100 ± 0.0	100 ± 0.0	75.4 ± 2.4
3.0	100 ± 0.0		90.2 ± 2.0			91.8 ± 6.3
4.5			100 ± 0.0			100 ± 0.0

* Concentrations of dried herbal material (µg/mL) in 5 mL saline water and the added corresponding extract volume (indicated in brackets) were the following: 9.8 (0.1); 45.5 (0.5); 83.3 (1.0); 142.9 (2.0); 187.5 (3.0) and 236.8 (4.5).

## Data Availability

Data are contained within the article and Supplementary Materials.

## Supplementary Materials

The following supporting information can be downloaded at https://www.mdpi.com/article/10.3390/plants15182851/s1. Figure S1: Geographical indication of sampling sites of *E. annuus* (L.) Pers. (Alytus County, Lazdijai district, Šlavantai) and *E. strigosus* Muhl. ex Willd. (Kaunas County, Prienai district, Būdvietis) in Lithuania; Figure S2: Images of *E. annuus* (L.) Pers. (a) and *E. strigosus* Muhl. ex Willd. (b) plants; Figure S3: Total ion chromatogram of *E. annuus* leaf extract in a positive (a) and negative (b) ionization mode; Figure S4: Total ion chromatogram of *E. strigosus* leaf extract in a positive (a) and negative (b) ionization mode; Figure S5: Gallic acid standard calibration curve, using Folin–Ciocalteu method; Figure S6: Calibration curves for AA determination by ABTS^●+^ scavenging assay; Figure S7: Calibration curves for AA determination by DPPH^●^ scavenging assay; Table S1: Total phenolic content (TPC, mg/L, expressed in gallic acid equivalent (GAE), *n *= 5, five replicates were conducted in total for the measurement) in aqueous extracts of *E. annuus* (L.) Pers. and *E. strigosus* Muhl. ex Willd. flowers, leaves and roots (plant material collected in 2024); Table S2: Total phenolic content (TPC, mg/L, expressed in gallic acid equivalent (GAE), *n *= 5) in aqueous and methanolic (MeOH:H2O, 1:1, *v*/*v*) extracts of *E. strigosus* Muhl. ex Willd. flowers, leaves and roots (plant material collected in 2025); Table S3: ABTS^●+^ and DPPH^●^ scavenging activity (mmol/L, TROLOX equivalent, *n *= 5, *n* indicates a number of measurements conducted using the same extract) of aqueous extracts from *E. annuus* (L.) Pers and *E. strigosus* Muhl. ex Willd. flowers, leaves and roots (plant material collected in 2024); Table S4: ABTS^●+^ and DPPH^●^ scavenging activities (TROLOX (mmol/L)) of aqueous and methanolic (1:1, *v*/*v*) extracts from *E. strigosus* Muhl. ex Willd. flowers, leaves and roots (plant material collected in 2025); Table S5: Chromatographic separation conditions of *Erigeron annuus* (L.) Pers. and *Erigeron strigosus* Muhl. ex Willd. extracts by gradient elution method (equilibration was for 5 min between runs; max pressure limit was 400 bar).

## Abbreviations

The following abbreviations are used in this manuscript:

HPLC/DAD/TOF	High-performance liquid chromatography/diode array detector/time of flight mass spectrometry
TROLOX	6-Hydroxy-2,5,7,8-tetramethylchroman-2-carboxylic acid (C_14_H_18_O_4_)
ABTS	2,2’-Azino-bis-(3-ethylbenzotiazoline-6-sulfonic acid) diammonium salt
DPPH	2,2-Diphenyl-1-picrylhydrazyl
GAE	Gallic acid equivalent
