# Supplementary material for: Evaluation of Phytochemistry, Toxicity and Radical-Scavenging Capacity of Two Erigeron Species (E. annuus (L.) Pers. and E. strigosus Muhl. ex Willd.) Extracts
Source: Plants (Basel). 2026 Sep 18;15(18):2851. doi: 10.3390/plants15182851 (PMC13610697; doi:10.3390/plants15182851)
Supplement: Supplementary file 1 [file plants-15-02851-s001.zip › plants-4512075-supplementary.pdf]

Evaluation of Phytochemistry, Toxicity and Radical Scavenging Capacity of Two *Erigeron* Species (*E. annuus* (L.) Pers. and *E. strigosus* Muhl. ex Willd.) Extracts

Asta Judžentienė and Jurga Būdienė

Table S1. Total phenolic content (TPC, mg/L, expressed in gallic acid equivalent (GAE), *n*=5, five replicates were conducted in total for the measurement) in aqueous extracts of *E. annuus* (L.) Pers. and *E. strigosus* Muhl. ex Willd. flowers, leaves and roots (plant material collected in 2024).

**TPC (mg/L, GAE) in aqueous extracts**			
	Flower	Leaf	Root
E. annuus	$81.88 \pm 2.96^{a*}$	$196.92 \pm 16.69^{b'}$	11.88 ± 1.27^{c}
E. strigosus	$102.05 \pm 9.16^{d**}$	$228.79 \pm 18.88^{e''}$	15.34 ± 2.08^{f}

Different superscript letters (a, b, c and d, e, f) indicate significant differences between TPC values of various plant organs within each species, whereas asterisks (*) and apostrophes (ʹ) demonstrate significant differences between *E. annuus* and *E. strigosus* within the same plant organ ($p \leq 0.05$, using the Wechs's t-test).

Table S2. Total phenolic content (TPC, mg/L, expressed in gallic acid equivalent (GAE), *n*=5) in aqueous and methanolic (MeOH:H_2O, 1:1, *v*/*v*) extracts of *E. strigosus* flowers, leaves and roots (plants collected in 2025).

**TPC (mg/L, GAE) in *E. strigosus* extracts**			
	Flower	Leaf	Root
Aqueous	$102.44 \pm 3.75^{a*}$	$239.36 \pm 5.77^{b'}$	17.17 ± 2.29^{c}
Methanolic	$113.36 \pm 4.19^{d**}$	$304.94 \pm 3.81^{e''}$	21.46 ± 4.33^{f}

Different superscript letters (a, b, c and d, e, f) indicate significant differences between the TPC values of various plant organs within the species, whereas asterisks and apostrophes show significant differences between *E. strigosus* extract types within the same plant organs ($p \leq 0.05$, by the Wechs's t-test).

Table S3. $ABTS^{\bullet+}$ and $DPPH^{\bullet}$ scavenging activity (mmol/L, TROLOX equivalent, *n*=5, *n* indicates a number of measurements conducted using the same extract) of aqueous extracts from *E. annuus* and *E. strigosus* flowers, leaves and roots (plant material collected in 2024).

**$ABTS^{\bullet+}$ Scavenging activity, TROLOX (mmol/L) of aqueous extracts**			
	Flower	Leaf	Root
E. annuus	$6.12 \pm 0.09^{a*}$	14.83 ± 0.25^{b}	0.17 ± 0.03^{c}
E. strigosus	$6.52 \pm 0.16^{d**}$	15.22 ± 0.13^{e}	0.16 ± 0.02^{f}
**$DPPH^{\bullet}$ Scavenging activity, TROLOX (mmol/L) of aqueous extracts**			
E. annuus	6.20 ± 0.08^{a}	$17.55 \pm 0.07^{b*}$	$3.06 \pm 0.03^{c'}$
E. strigosus	6.08 ± 0.04^{d}	$16.45 \pm 0.04^{e**}$	$3.33 \pm 0.10^{f''}$

Different superscript letters demonstrate significant differences among the $ABTS^{\bullet+}$ and $DPPH^{\bullet}$ scavenging activity values of various plant organs within each species, whereas asterisks and apostrophes indicate significant differences between *E. annuus* and *E. strigosus* within the same plant organs ($p \leq 0.05$, using the Wechs's t-test).

Table S4. ABTS$^{•+}$ and DPPH$^{•}$ scavenging activities (TROLOX (mmol/L), n=5) of aqueous and methanolic (1:1, v/v) extracts from *E. strigosus* flowers, leaves and roots (plant material collected in 2025).

**ABTS$^{•+}$ Scavenging activity, TROLOX (mmol/L) of *E. strigosus* extracts**			
	Flower	Leaf	Root
Aqueous	5.91 ± 0.31^{a}	$13.96 \pm 0.39^{b*}$	$0.24 \pm 0.00^{c'}$
Methanolic	6.60 ± 0.32^{d}	$17.15 \pm 0.43^{e**}$	$0.67 \pm 0.01^{f''}$
**DPPH$^{•}$ Scavenging activity, TROLOX (mmol/L) of *E. strigosus* extracts**			
Aqueous	5.53 ± 0.25^{a}	16.26 ± 0.66^{b}	$2.83 \pm 0.01^{c*}$
Methanolic	5.96 ± 0.80^{d}	17.50 ± 0.20^{e}	$3.29 \pm 0.01^{f*}$

Different superscript letters show significant differences among the ABTS$^{•+}$ and DPPH$^{•}$ scavenging capacity values of various plant organs within each species, whereas asterisks indicate significant differences between *E. strigosus* extract types within the same plant organ (column-wise comparison) ($p \leq 0.05$).

Table S5. Chromatographic separation conditions of *Erigeron annuus* (L.) Pers. and *Erigeron strigosus* Muhl. ex Willd. extracts by gradient elution method (equilibration was for 5 min between runs, max pressure limit was set 400 bars).

**Time, min**	**A, %**	**B, %**
0 (initial)	95	5
25	0	100
28 (from 25 min isocratic mode)	0	100
28.1	95	5

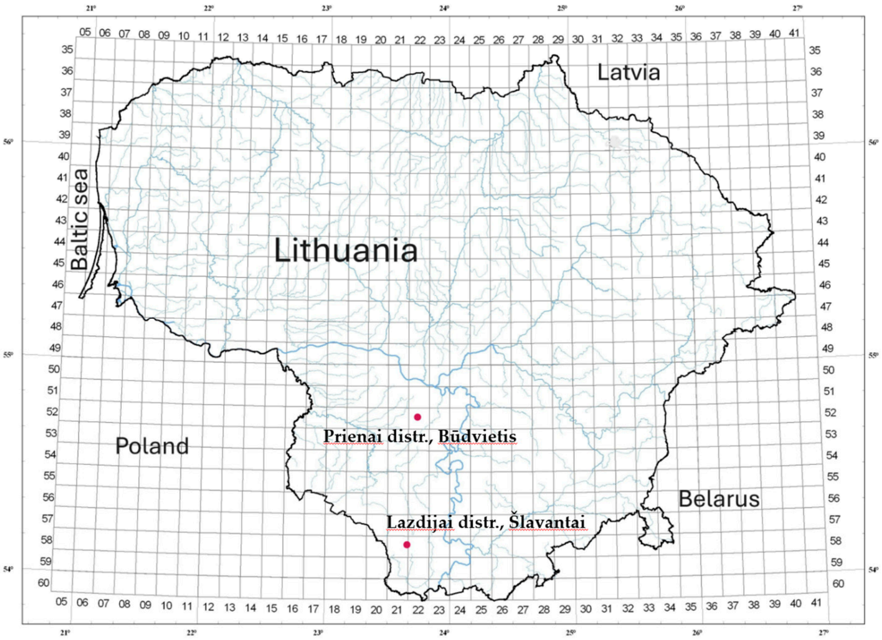

Figure S1. Geographical indication of sampling sites of *E. annuus* (L.) Pers. (Alytus County, Lazdijai district, Šlavantai) and *E. strigosus* Muhl. ex Willd. (Kaunas County, Prienai district, Būdvietis) in Lithuania.

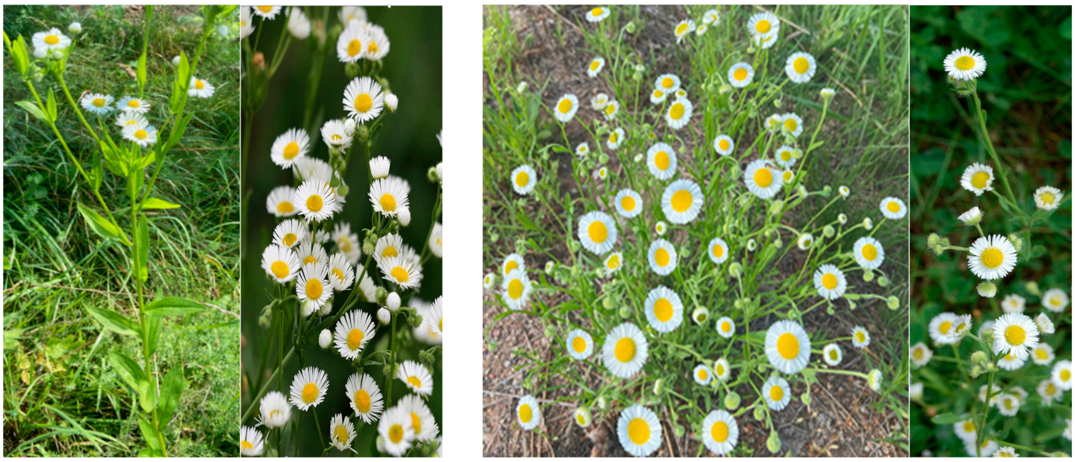

(**a**) (**b**)

Figure S2. Imagines of *E. annuus* (L.) Pers. (a) and *E. strigosus* Muhl. ex Willd. (b) plants.

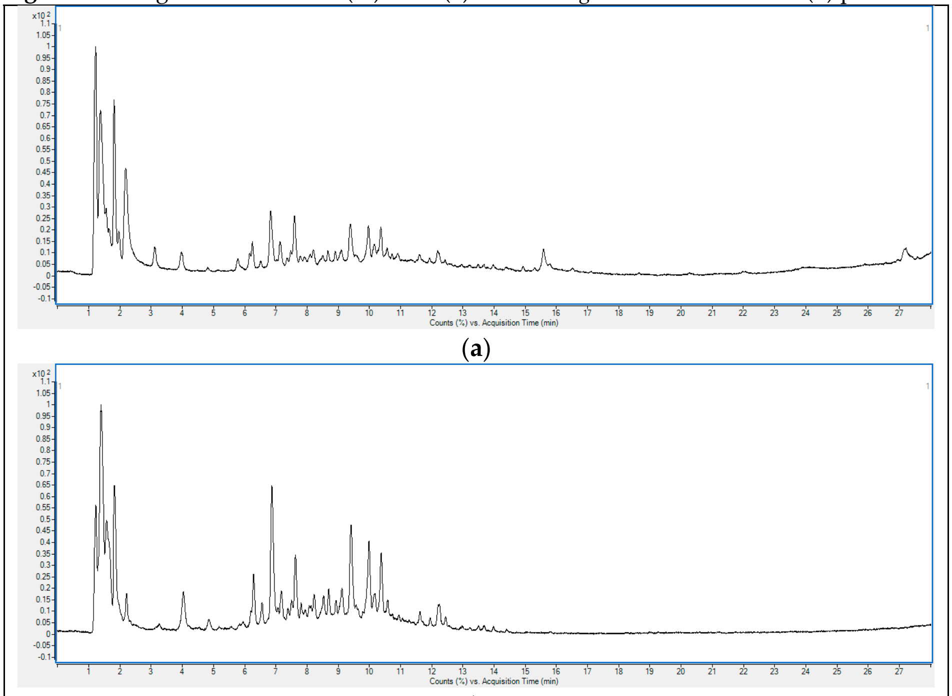

(**a**)

(**b**)

Figure S3. Total ion chromatogram of *E. annuus* leaf extract in a positive (a) and negative (b) ionization mode.

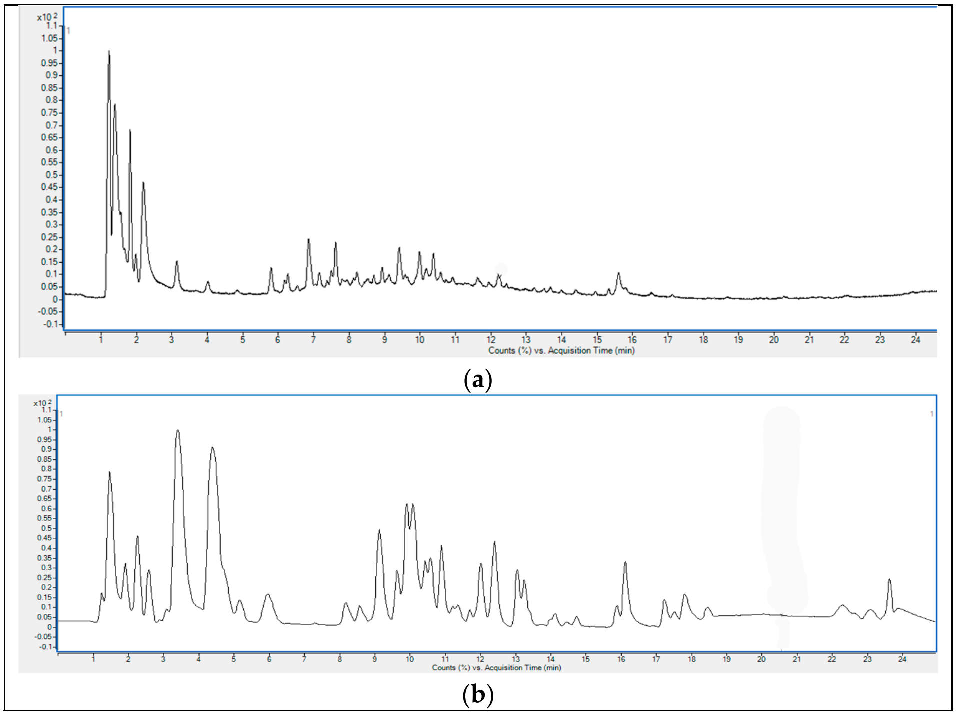

Figure S4. Total ion chromatogram of *E. strigosus* leaf extract in a positive (a) and negative (b) ionization mode.

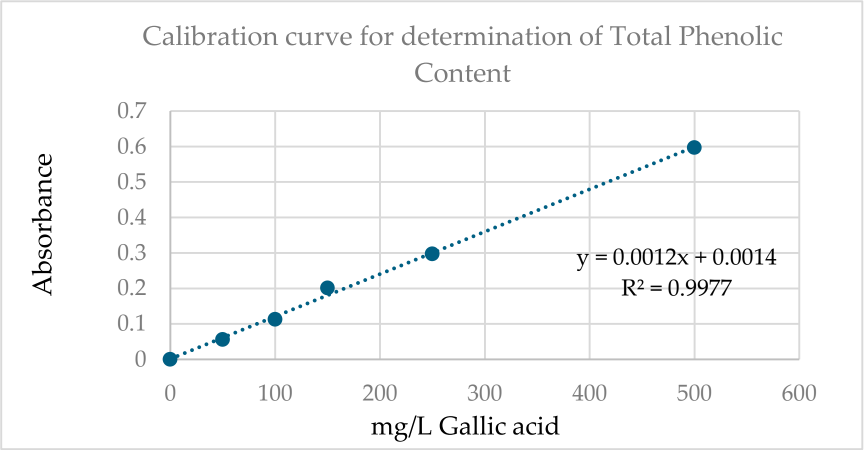

Figure S5. Gallic acid standard calibration curve, using Folin-Ciocalteu method.

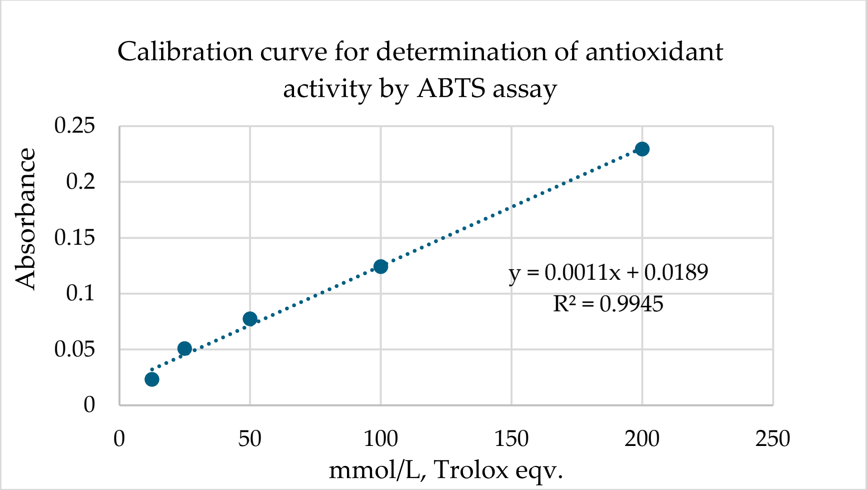

Figure S6. Calibration curve for AA determination by ABTS$^{•+}$ scavenging assay.

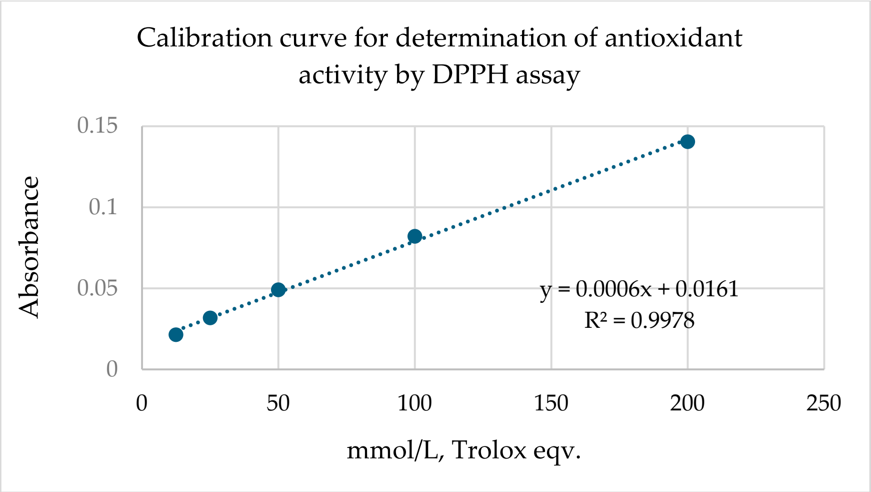

Figure S7. Calibration curve for AA determination by DPPH$^{•}$ scavenging assay.
